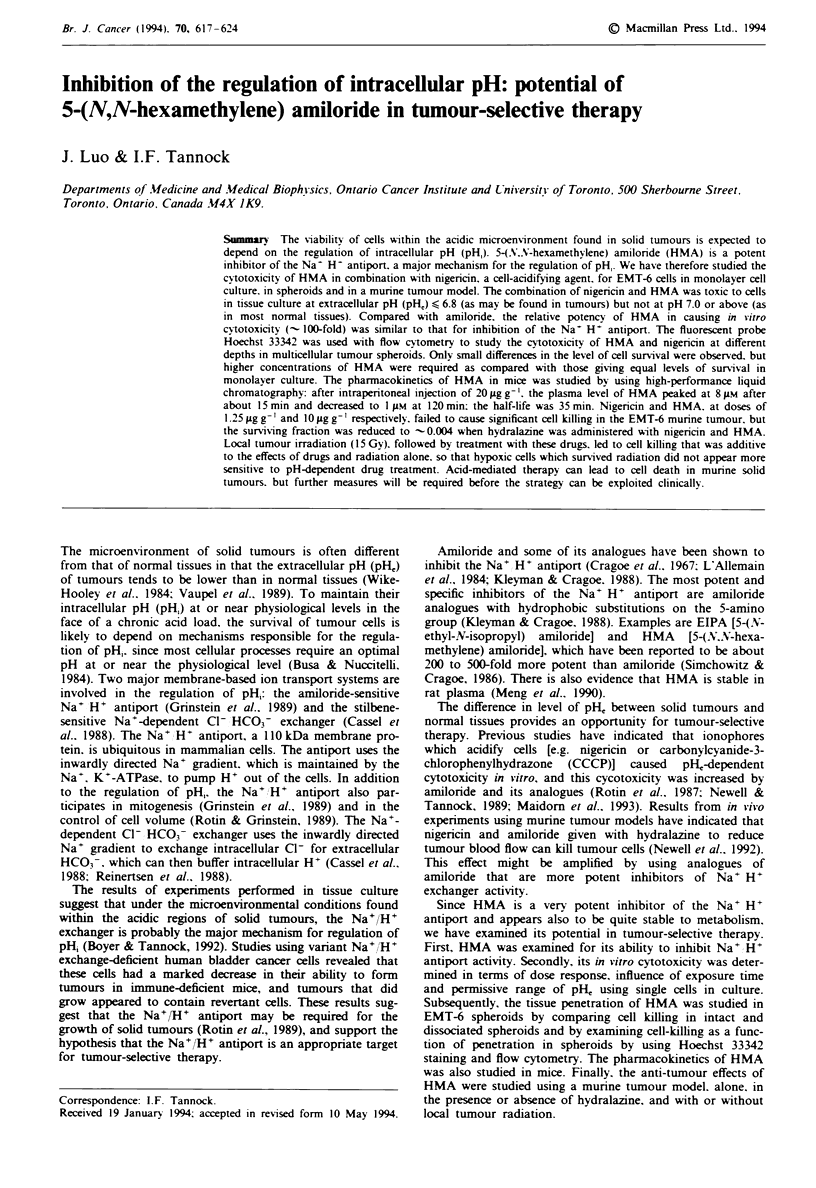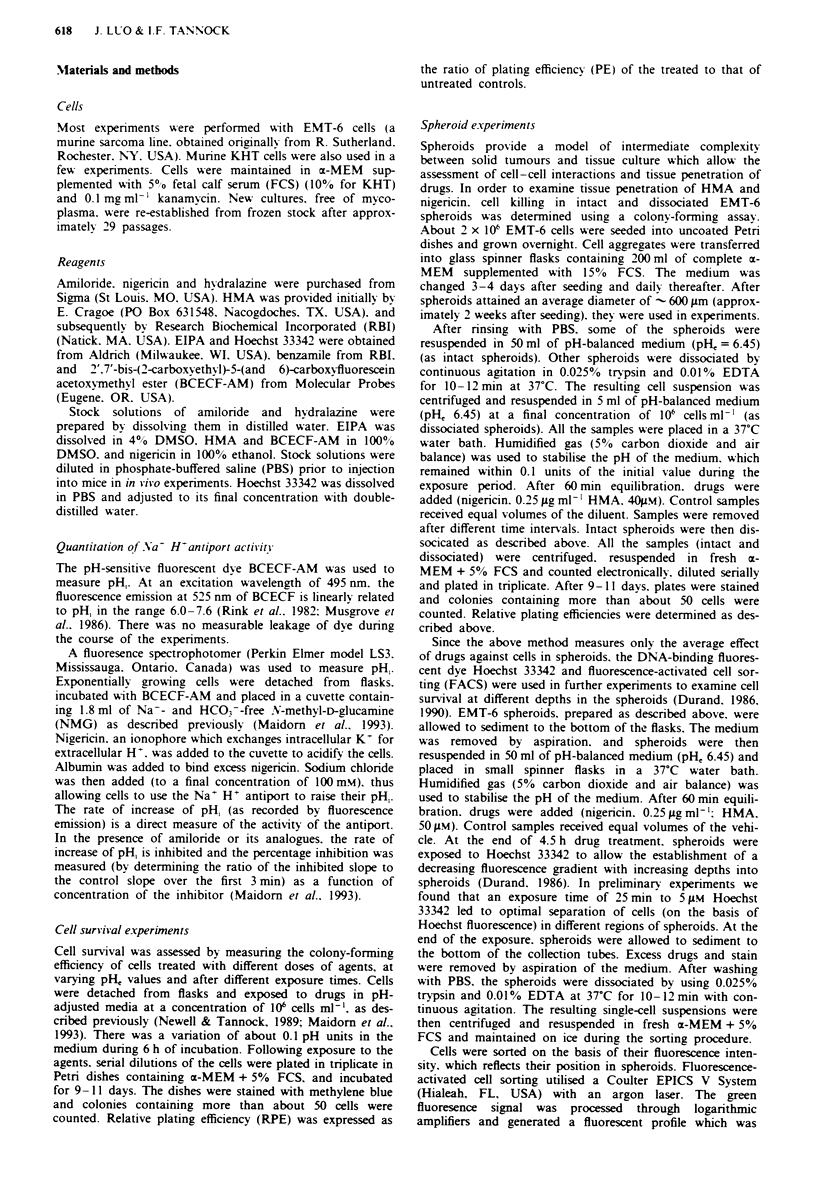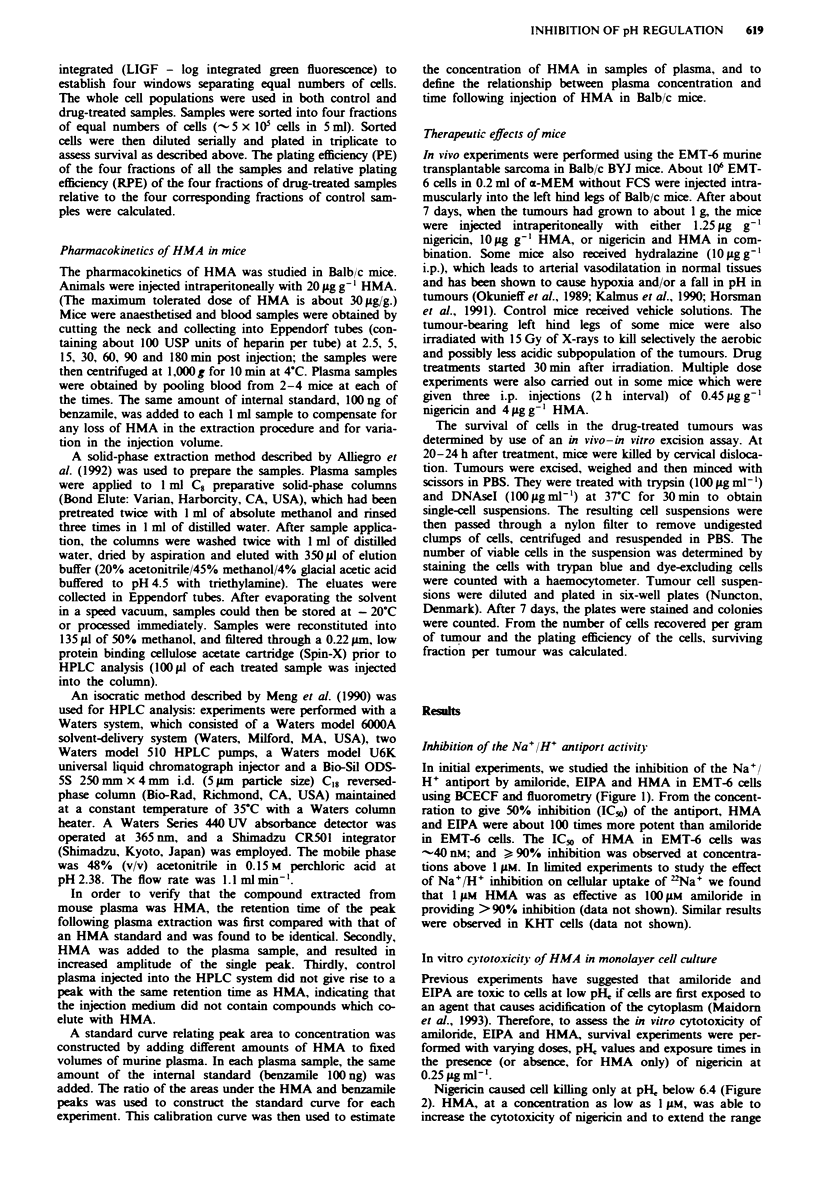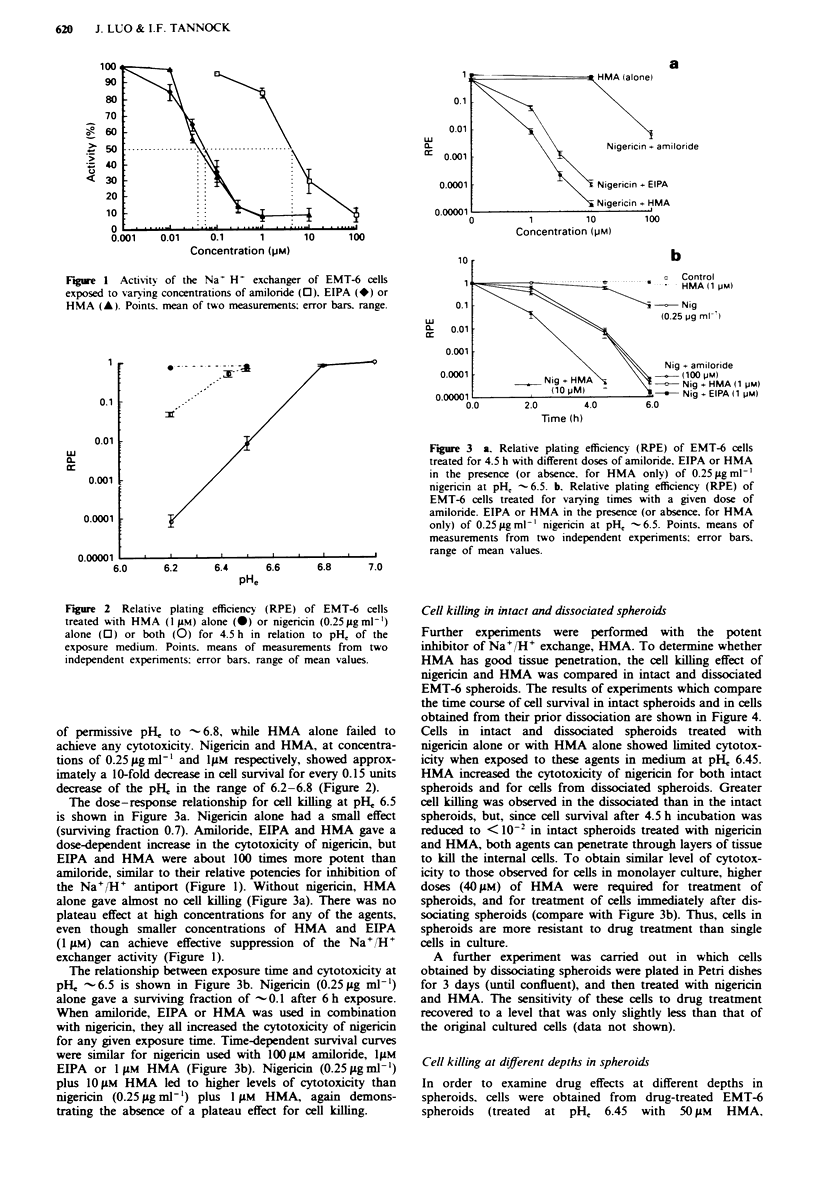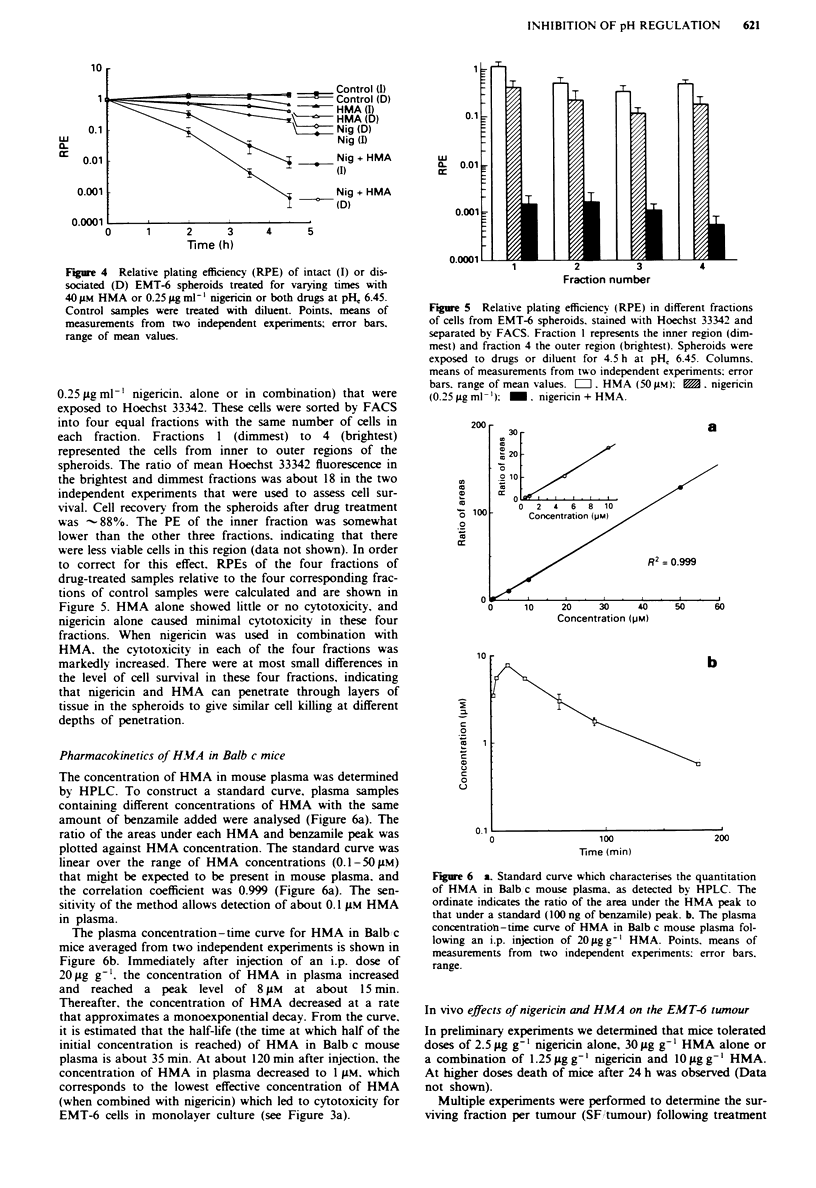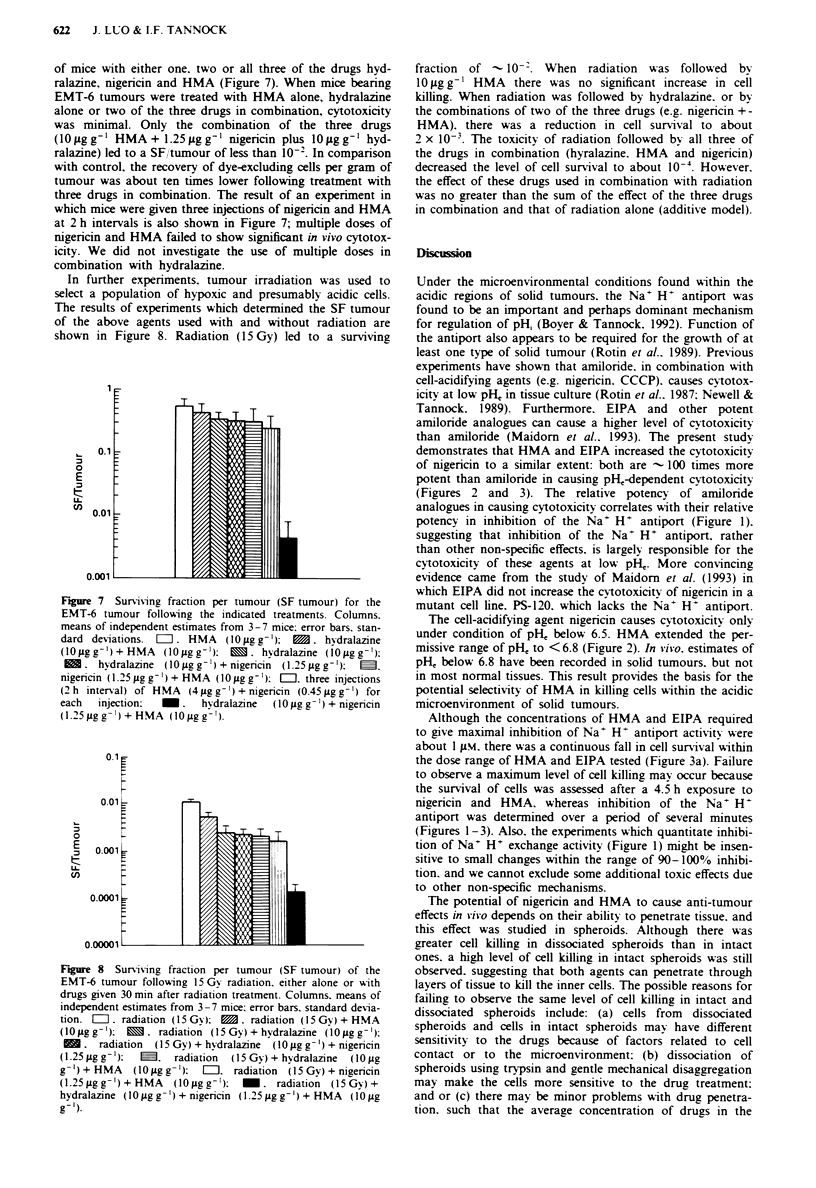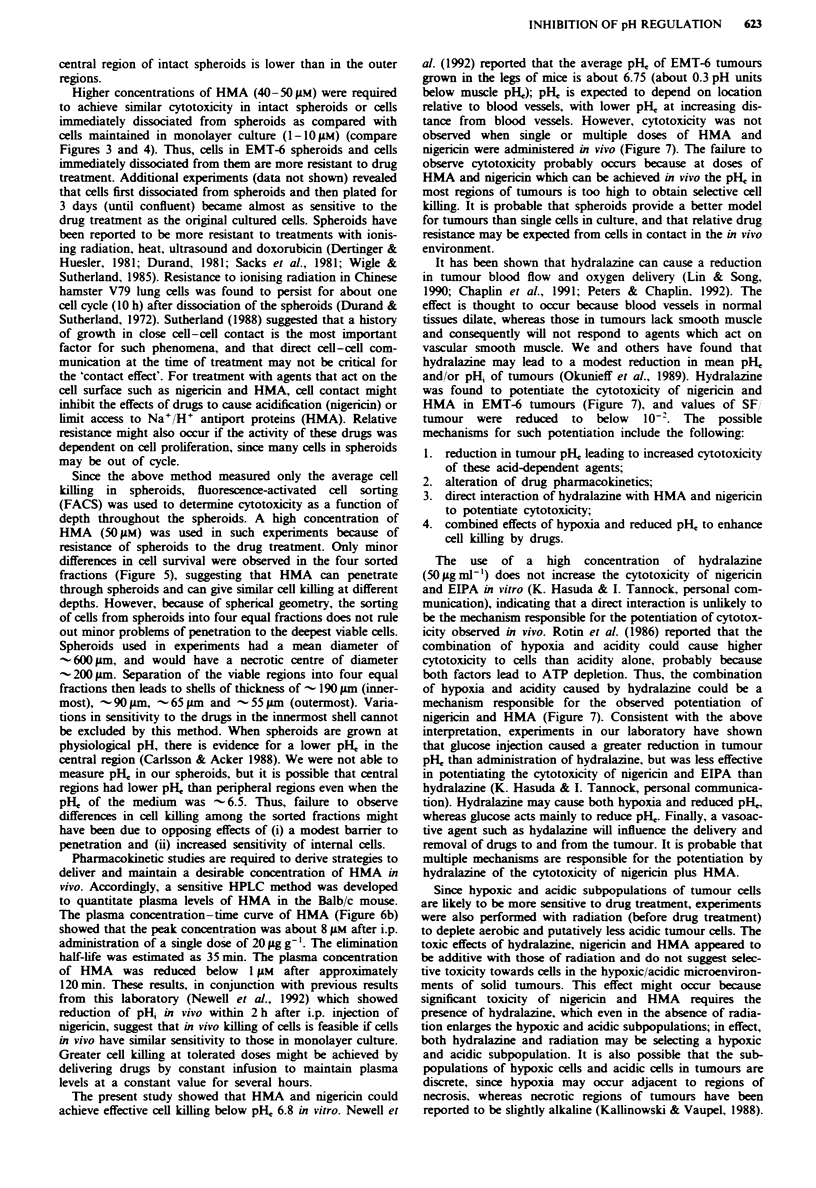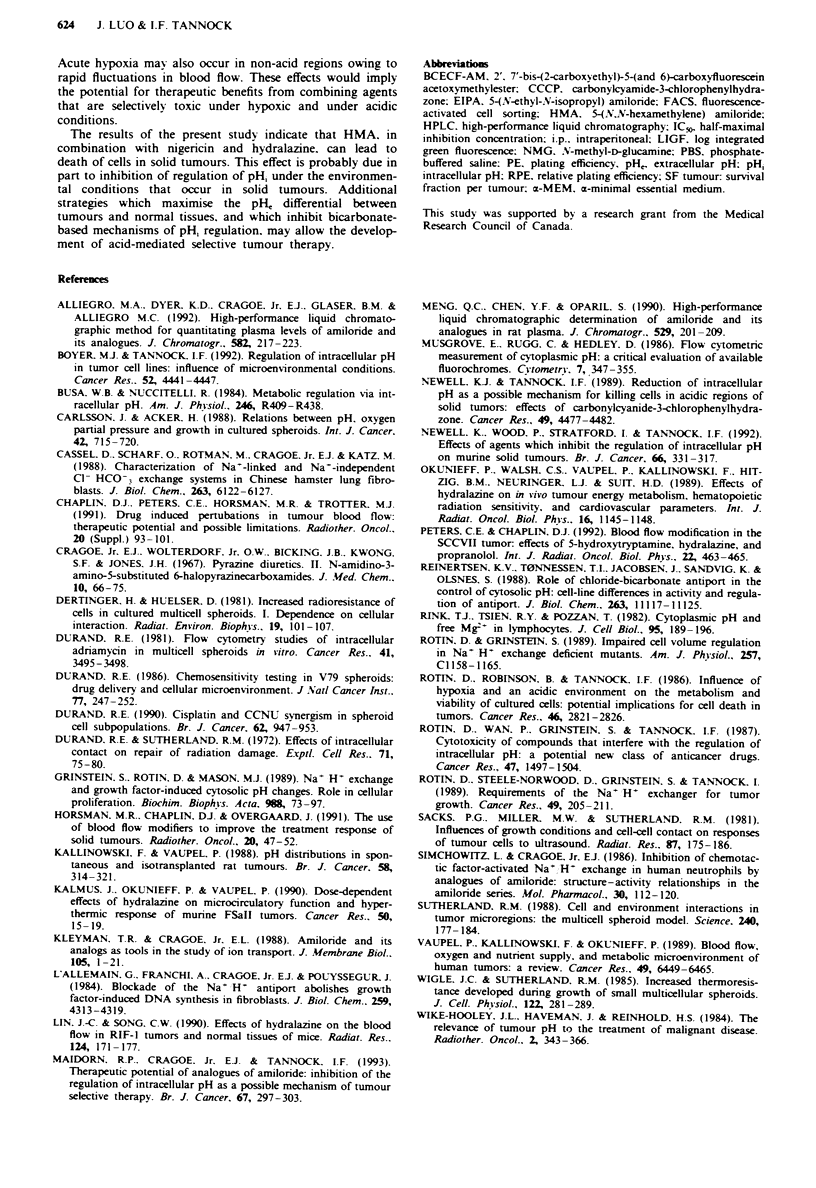# Inhibition of the regulation of intracellular pH: potential of 5-(N,N-hexamethylene) amiloride in tumour-selective therapy.

**DOI:** 10.1038/bjc.1994.360

**Published:** 1994-10

**Authors:** J. Luo, I. F. Tannock

**Affiliations:** Department of Medicine, Ontario Cancer Institute, Toronto, Canada.

## Abstract

The viability of cells within the acidic microenvironment found in solid tumours is expected to depend on the regulation of intracellular pH (pHi). 5-(N,N-hexamethylene) amiloride (HMA) is a potent inhibitor of the Na+/H+ antiport, a major mechanism for the regulation of pHi. We have therefore studied the cytotoxicity of HMA in combination with nigericin, a cell-acidifying agent, for EMT-6 cells in monolayer cell culture, in spheroids and in a murine tumour model. The combination of nigericin and HMA was toxic to cells in tissue culture at extracellular pH (pHe) < or = 6.8 (as may be found in tumours) but not at pH 7.0 or above (as in most normal tissues). Compared with amiloride, the relative potency of HMA in causing in vitro cytotoxicity (approximately 100-fold) was similar to that for inhibition of the Na+/H+ antiport. The fluorescent probe Hoechst 33342 was used with flow cytometry to study the cytotoxicity of HMA and nigericin at different depths in multicellular tumour spheroids. Only small differences in the level of cell survival were observed, but higher concentrations of HMA were required as compared with those giving equal levels of survival in monolayer culture. The pharmacokinetics of HMA in mice was studied by using high-performance liquid chromatography: after intraperitoneal injection of 20 micrograms g-1, the plasma level of HMA peaked at 8 microM after about 15 min and decreased to 1 microM at 120 min; the half-life was 35 min. Nigericin and HMA, at doses of 1.25 micrograms g-1 and 10 micrograms g-1 respectively, failed to cause significant cell killing in the EMT-6 murine tumour, but the surviving fraction was reduced to approximately 0.004 when hydralazine was administered with nigericin and HMA. Local tumour irradiation (15 Gy), followed by treatment with these drugs, led to cell killing that was additive to the effects of drugs and radiation alone, so that hypoxic cells which survived radiation did not appear more sensitive to pH-dependent drug treatment. Acid-mediated therapy can lead to cell death in murine solid tumours, but further measures will be required before the strategy can be exploited clinically.


Br. J. Cancer (1994), 70, 617–624 © Macmillan Press Ltd., 1994

### Inhibition of the regulation of intracellular pH: potential of 5-(*N*,*N*-hexamethylene) amiloride in tumour-selective therapy

J. Luo & I.F. Tannock

*Departments of Medicine and Medical Biophysics, Ontario Cancer Institute and University of Toronto, 500 Sherbourne Street, Toronto, Ontario, Canada M4X 1K9.*

**Summary** The viability of cells within the acidic microenvironment found in solid tumours is expected to depend on the regulation of intracellular pH ($pH_i$). 5-(*N*,*N*-hexamethylene) amiloride (HMA) is a potent inhibitor of the $Na^+/H^+$ antiport, a major mechanism for the regulation of $pH_i$. We have therefore studied the cytotoxicity of HMA in combination with nigericin, a cell-acidifying agent, for EMT-6 cells in monolayer cell culture, in spheroids and in a murine tumour model. The combination of nigericin and HMA was toxic to cells in tissue culture at extracellular pH ($pH_e$) $\leqslant 6.8$ (as may be found in tumours) but not at pH 7.0 or above (as in most normal tissues). Compared with amiloride, the relative potency of HMA in causing *in vitro* cytotoxicity ($\sim$100-fold) was similar to that for inhibition of the $Na^+/H^+$ antiport. The fluorescent probe Hoechst 33342 was used with flow cytometry to study the cytotoxicity of HMA and nigericin at different depths in multicellular tumour spheroids. Only small differences in the level of cell survival were observed, but higher concentrations of HMA were required as compared with those giving equal levels of survival in monolayer culture. The pharmacokinetics of HMA in mice was studied by using high-performance liquid chromatography: after intraperitoneal injection of 20 $\mu g\ g^{-1}$, the plasma level of HMA peaked at 8 $\mu$M after about 15 min and decreased to 1 $\mu$M at 120 min; the half-life was 35 min. Nigericin and HMA, at doses of 1.25 $\mu g\ g^{-1}$ and 10 $\mu g\ g^{-1}$ respectively, failed to cause significant cell killing in the EMT-6 murine tumour, but the surviving fraction was reduced to $\sim$0.004 when hydralazine was administered with nigericin and HMA. Local tumour irradiation (15 Gy), followed by treatment with these drugs, led to cell killing that was additive to the effects of drugs and radiation alone, so that hypoxic cells which survived radiation did not appear more sensitive to pH-dependent drug treatment. Acid-mediated therapy can lead to cell death in murine solid tumours, but further measures will be required before the strategy can be exploited clinically.

The microenvironment of solid tumours is often different from that of normal tissues in that the extracellular pH ($pH_e$) of tumours tends to be lower than in normal tissues (Wike-Hooley *et al.*, 1984; Vaupel *et al.*, 1989). To maintain their intracellular pH ($pH_i$) at or near physiological levels in the face of a chronic acid load, the survival of tumour cells is likely to depend on mechanisms responsible for the regulation of $pH_i$, since most cellular processes require an optimal pH at or near the physiological level (Busa & Nuccitelli, 1984). Two major membrane-based ion transport systems are involved in the regulation of $pH_i$: the amiloride-sensitive $Na^+/H^+$ antiport (Grinstein *et al.*, 1989) and the stilbene-sensitive $Na^+$-dependent $Cl^-/HCO_3^-$ exchanger (Cassel *et al.*, 1988). The $Na^+/H^+$ antiport, a 110 kDa membrane protein, is ubiquitous in mammalian cells. The antiport uses the inwardly directed $Na^+$ gradient, which is maintained by the $Na^+$, $K^+$-ATPase, to pump $H^+$ out of the cells. In addition to the regulation of $pH_i$, the $Na^+/H^+$ antiport also participates in mitogenesis (Grinstein *et al.*, 1989) and in the control of cell volume (Rotin & Grinstein, 1989). The $Na^+$-dependent $Cl^-/HCO_3^-$ exchanger uses the inwardly directed $Na^+$ gradient to exchange intracellular $Cl^-$ for extracellular $HCO_3^-$, which can then buffer intracellular $H^+$ (Cassel *et al.*, 1988; Reinertsen *et al.*, 1988).

The results of experiments performed in tissue culture suggest that under the microenvironmental conditions found within the acidic regions of solid tumours, the $Na^+/H^+$ exchanger is probably the major mechanism for regulation of $pH_i$ (Boyer & Tannock, 1992). Studies using variant $Na^+/H^+$ exchange-deficient human bladder cancer cells revealed that these cells had a marked decrease in their ability to form tumours in immune-deficient mice, and tumours that did grow appeared to contain revertant cells. These results suggest that the $Na^+/H^+$ antiport may be required for the growth of solid tumours (Rotin *et al.*, 1989), and support the hypothesis that the $Na^+/H^+$ antiport is an appropriate target for tumour-selective therapy.

Amiloride and some of its analogues have been shown to inhibit the $Na^+/H^+$ antiport (Cragoe *et al.*, 1967; L'Allemain *et al.*, 1984; Kleyman & Cragoe, 1988). The most potent and specific inhibitors of the $Na^+/H^+$ antiport are amiloride analogues with hydrophobic substitutions on the 5-amino group (Kleyman & Cragoe, 1988). Examples are EIPA [5-(*N*-ethyl-*N*-isopropyl) amiloride] and HMA [5-(*N*,*N*-hexamethylene) amiloride], which have been reported to be about 200 to 500-fold more potent than amiloride (Simchowitz & Cragoe, 1986). There is also evidence that HMA is stable in rat plasma (Meng *et al.*, 1990).

The difference in level of $pH_e$ between solid tumours and normal tissues provides an opportunity for tumour-selective therapy. Previous studies have indicated that ionophores which acidify cells [e.g. nigericin or carbonylcyanide-3-chlorophenylhydrazone (CCCP)] caused $pH_e$-dependent cytotoxicity *in vitro*, and this cycotoxicity was increased by amiloride and its analogues (Rotin *et al.*, 1987; Newell & Tannock, 1989; Maidorn *et al.*, 1993). Results from *in vivo* experiments using murine tumour models have indicated that nigericin and amiloride given with hydralazine to reduce tumour blood flow can kill tumour cells (Newell *et al.*, 1992). This effect might be amplified by using analogues of amiloride that are more potent inhibitors of $Na^+/H^+$ exchanger activity.

Since HMA is a very potent inhibitor of the $Na^+/H^+$ antiport and appears also to be quite stable to metabolism, we have examined its potential in tumour-selective therapy. First, HMA was examined for its ability to inhibit $Na^+/H^+$ antiport activity. Secondly, its *in vitro* cytotoxicity was determined in terms of dose response, influence of exposure time and permissive range of $pH_e$ using single cells in culture. Subsequently, the tissue penetration of HMA was studied in EMT-6 spheroids by comparing cell killing in intact and dissociated spheroids and by examining cell-killing as a function of penetration in spheroids by using Hoechst 33342 staining and flow cytometry. The pharmacokinetics of HMA was also studied in mice. Finally, the anti-tumour effects of HMA were studied using a murine tumour model, alone, in the presence or absence of hydralazine, and with or without local tumour radiation.

Correspondence: I.F. Tannock.
Received 19 January 1994; accepted in revised form 10 May 1994.

### Materials and methods

### *Cells*

Most experiments were performed with EMT-6 cells (a murine sarcoma line, obtained originally from R. Sutherland, Rochester, NY, USA). Murine KHT cells were also used in a few experiments. Cells were maintained in α-MEM supplemented with 5% fetal calf serum (FCS) (10% for KHT) and 0.1 mg ml$^{-1}$ kanamycin. New cultures, free of mycoplasma, were re-established from frozen stock after approximately 29 passages.

### *Reagents*

Amiloride, nigericin and hydralazine were purchased from Sigma (St Louis, MO, USA). HMA was provided initially by E. Cragoe (PO Box 631548, Nacogdoches, TX, USA), and subsequently by Research Biochemical Incorporated (RBI) (Natick, MA, USA). EIPA and Hoechst 33342 were obtained from Aldrich (Milwaukee, WI, USA), benzamile from RBI, and 2',7'-bis-(2-carboxyethyl)-5-(and 6)-carboxyfluorescein acetoxymethyl ester (BCECF-AM) from Molecular Probes (Eugene, OR, USA).

Stock solutions of amiloride and hydralazine were prepared by dissolving them in distilled water. EIPA was dissolved in 4% DMSO, HMA and BCECF-AM in 100% DMSO, and nigericin in 100% ethanol. Stock solutions were diluted in phosphate-buffered saline (PBS) prior to injection into mice in *in vivo* experiments. Hoechst 33342 was dissolved in PBS and adjusted to its final concentration with double-distilled water.

### *Quantitation of $Na^+$ $H^+$ antiport activity*

The pH-sensitive fluorescent dye BCECF-AM was used to measure $pH_i$. At an excitation wavelength of 495 nm, the fluorescence emission at 525 nm of BCECF is linearly related to $pH_i$ in the range 6.0–7.6 (Rink *et al.*, 1982; Musgrove *et al.*, 1986). There was no measurable leakage of dye during the course of the experiments.

A fluoresence spectrophotomer (Perkin Elmer model LS3, Mississauga, Ontario, Canada) was used to measure $pH_i$. Exponentially growing cells were detached from flasks, incubated with BCECF-AM and placed in a cuvette containing 1.8 ml of $Na^+$- and $HCO_3^-$-free *N*-methyl-D-glucamine (NMG) as described previously (Maidorn *et al.*, 1993). Nigericin, an ionophore which exchanges intracellular $K^+$ for extracellular $H^+$, was added to the cuvette to acidify the cells. Albumin was added to bind excess nigericin. Sodium chloride was then added (to a final concentration of 100 mM), thus allowing cells to use the $Na^+$ $H^+$ antiport to raise their $pH_i$. The rate of increase of $pH_i$ (as recorded by fluorescence emission) is a direct measure of the activity of the antiport. In the presence of amiloride or its analogues, the rate of increase of $pH_i$ is inhibited and the percentage inhibition was measured (by determining the ratio of the inhibited slope to the control slope over the first 3 min) as a function of concentration of the inhibitor (Maidorn *et al.*, 1993).

### *Cell survival experiments*

Cell survival was assessed by measuring the colony-forming efficiency of cells treated with different doses of agents, at varying $pH_e$ values and after different exposure times. Cells were detached from flasks and exposed to drugs in pH-adjusted media at a concentration of $10^6$ cells ml$^{-1}$, as described previously (Newell & Tannock, 1989; Maidorn *et al.*, 1993). There was a variation of about 0.1 pH units in the medium during 6 h of incubation. Following exposure to the agents, serial dilutions of the cells were plated in triplicate in Petri dishes containing α-MEM + 5% FCS, and incubated for 9–11 days. The dishes were stained with methylene blue and colonies containing more than about 50 cells were counted. Relative plating efficiency (RPE) was expressed as the ratio of plating efficiency (PE) of the treated to that of untreated controls.

### *Spheroid experiments*

Spheroids provide a model of intermediate complexity between solid tumours and tissue culture which allow the assessment of cell–cell interactions and tissue penetration of drugs. In order to examine tissue penetration of HMA and nigericin, cell killing in intact and dissociated EMT-6 spheroids was determined using a colony-forming assay. About 2 × $10^6$ EMT-6 cells were seeded into uncoated Petri dishes and grown overnight. Cell aggregates were transferred into glass spinner flasks containing 200 ml of complete α-MEM supplemented with 15% FCS. The medium was changed 3–4 days after seeding and daily thereafter. After spheroids attained an average diameter of ~600 μm (approximately 2 weeks after seeding), they were used in experiments.

After rinsing with PBS, some of the spheroids were resuspended in 50 ml of pH-balanced medium ($pH_e$ = 6.45) (as intact spheroids). Other spheroids were dissociated by continuous agitation in 0.025% trypsin and 0.01% EDTA for 10–12 min at 37°C. The resulting cell suspension was centrifuged and resuspended in 5 ml of pH-balanced medium ($pH_e$ 6.45) at a final concentration of $10^6$ cells ml$^{-1}$ (as dissociated spheroids). All the samples were placed in a 37°C water bath. Humidified gas (5% carbon dioxide and air balance) was used to stabilise the pH of the medium, which remained within 0.1 units of the initial value during the exposure period. After 60 min equilibration, drugs were added (nigericin, 0.25 μg ml$^{-1}$ HMA, 40μM). Control samples received equal volumes of the diluent. Samples were removed after different time intervals. Intact spheroids were then dissocicated as described above. All the samples (intact and dissociated) were centrifuged, resuspended in fresh α-MEM + 5% FCS and counted electronically, diluted serially and plated in triplicate. After 9–11 days, plates were stained and colonies containing more than about 50 cells were counted. Relative plating efficiencies were determined as described above.

Since the above method measures only the average effect of drugs against cells in spheroids, the DNA-binding fluorescent dye Hoechst 33342 and fluorescence-activated cell sorting (FACS) were used in further experiments to examine cell survival at different depths in the spheroids (Durand, 1986, 1990). EMT-6 spheroids, prepared as described above, were allowed to sediment to the bottom of the flasks. The medium was removed by aspiration, and spheroids were then resuspended in 50 ml of pH-balanced medium ($pH_e$ 6.45) and placed in small spinner flasks in a 37°C water bath. Humidified gas (5% carbon dioxide and air balance) was used to stabilise the pH of the medium. After 60 min equilibration, drugs were added (nigericin, 0.25 μg ml$^{-1}$; HMA, 50 μM). Control samples received equal volumes of the vehicle. At the end of 4.5 h drug treatment, spheroids were exposed to Hoechst 33342 to allow the establishment of a decreasing fluorescence gradient with increasing depths into spheroids (Durand, 1986). In preliminary experiments we found that an exposure time of 25 min to 5 μM Hoechst 33342 led to optimal separation of cells (on the basis of Hoechst fluorescence) in different regions of spheroids. At the end of the exposure, spheroids were allowed to sediment to the bottom of the collection tubes. Excess drugs and stain were removed by aspiration of the medium. After washing with PBS, the spheroids were dissociated by using 0.025% trypsin and 0.01% EDTA at 37°C for 10–12 min with continuous agitation. The resulting single-cell suspensions were then centrifuged and resuspended in fresh α-MEM + 5% FCS and maintained on ice during the sorting procedure.

Cells were sorted on the basis of their fluorescence intensity, which reflects their position in spheroids. Fluorescence-activated cell sorting utilised a Coulter EPICS V System (Hialeah, FL, USA) with an argon laser. The green fluoresence signal was processed through logarithmic amplifiers and generated a fluorescent profile which was integrated (LIGF - log integrated green fluorescence) to establish four windows separating equal numbers of cells. The whole cell populations were used in both control and drug-treated samples. Samples were sorted into four fractions of equal numbers of cells ($\sim 5 \times 10^5$ cells in 5 ml). Sorted cells were then diluted serially and plated in triplicate to assess survival as described above. The plating efficiency (PE) of the four fractions of all the samples and relative plating efficiency (RPE) of the four fractions of drug-treated samples relative to the four corresponding fractions of control samples were calculated.

### *Pharmacokinetics of HMA in mice*

The pharmacokinetics of HMA was studied in Balb/c mice. Animals were injected intraperitoneally with 20 $\mu g\ g^{-1}$ HMA. (The maximum tolerated dose of HMA is about 30 $\mu g/g$.) Mice were anaesthetised and blood samples were obtained by cutting the neck and collecting into Eppendorf tubes (containing about 100 USP units of heparin per tube) at 2.5, 5, 15, 30, 60, 90 and 180 min post injection; the samples were then centrifuged at 1,000 *g* for 10 min at 4°C. Plasma samples were obtained by pooling blood from 2–4 mice at each of the times. The same amount of internal standard, 100 ng of benzamile, was added to each 1 ml sample to compensate for any loss of HMA in the extraction procedure and for variation in the injection volume.

A solid-phase extraction method described by Alliegro *et al.* (1992) was used to prepare the samples. Plasma samples were applied to 1 ml $C_8$ preparative solid-phase columns (Bond Elute: Varian, Harborcity, CA, USA), which had been pretreated twice with 1 ml of absolute methanol and rinsed three times in 1 ml of distilled water. After sample application, the columns were washed twice with 1 ml of distilled water, dried by aspiration and eluted with 350 $\mu l$ of elution buffer (20% acetonitrile/45% methanol/4% glacial acetic acid buffered to pH 4.5 with triethylamine). The eluates were collected in Eppendorf tubes. After evaporating the solvent in a speed vacuum, samples could then be stored at $-20°C$ or processed immediately. Samples were reconstituted into 135 $\mu l$ of 50% methanol, and filtered through a 0.22 $\mu m$, low protein binding cellulose acetate cartridge (Spin-X) prior to HPLC analysis (100 $\mu l$ of each treated sample was injected into the column).

An isocratic method described by Meng *et al.* (1990) was used for HPLC analysis: experiments were performed with a Waters system, which consisted of a Waters model 6000A solvent-delivery system (Waters, Milford, MA, USA), two Waters model 510 HPLC pumps, a Waters model U6K universal liquid chromatograph injector and a Bio-Sil ODS-5S 250 mm × 4 mm i.d. (5 $\mu m$ particle size) $C_{18}$ reversed-phase column (Bio-Rad, Richmond, CA, USA) maintained at a constant temperature of 35°C with a Waters column heater. A Waters Series 440 UV absorbance detector was operated at 365 nm, and a Shimadzu CR501 integrator (Shimadzu, Kyoto, Japan) was employed. The mobile phase was 48% (v/v) acetonitrile in 0.15 M perchloric acid at pH 2.38. The flow rate was 1.1 ml $min^{-1}$.

In order to verify that the compound extracted from mouse plasma was HMA, the retention time of the peak following plasma extraction was first compared with that of an HMA standard and was found to be identical. Secondly, HMA was added to the plasma sample, and resulted in increased amplitude of the single peak. Thirdly, control plasma injected into the HPLC system did not give rise to a peak with the same retention time as HMA, indicating that the injection medium did not contain compounds which co-elute with HMA.

A standard curve relating peak area to concentration was constructed by adding different amounts of HMA to fixed volumes of murine plasma. In each plasma sample, the same amount of the internal standard (benzamile 100 ng) was added. The ratio of the areas under the HMA and benzamile peaks was used to construct the standard curve for each experiment. This calibration curve was then used to estimate the concentration of HMA in samples of plasma, and to define the relationship between plasma concentration and time following injection of HMA in Balb/c mice.

### *Therapeutic effects of mice*

*In vivo* experiments were performed using the EMT-6 murine transplantable sarcoma in Balb/c BYJ mice. About $10^6$ EMT-6 cells in 0.2 ml of α-MEM without FCS were injected intramuscularly into the left hind legs of Balb/c mice. After about 7 days, when the tumours had grown to about 1 g, the mice were injected intraperitoneally with either 1.25 $\mu g\ g^{-1}$ nigericin, 10 $\mu g\ g^{-1}$ HMA, or nigericin and HMA in combination. Some mice also received hydralazine (10 $\mu g\ g^{-1}$ i.p.), which leads to arterial vasodilatation in normal tissues and has been shown to cause hypoxia and/or a fall in pH in tumours (Okunieff *et al.*, 1989; Kalmus *et al.*, 1990; Horsman *et al.*, 1991). Control mice received vehicle solutions. The tumour-bearing left hind legs of some mice were also irradiated with 15 Gy of X-rays to kill selectively the aerobic and possibly less acidic subpopulation of the tumours. Drug treatments started 30 min after irradiation. Multiple dose experiments were also carried out in some mice which were given three i.p. injections (2 h interval) of 0.45 $\mu g\ g^{-1}$ nigericin and 4 $\mu g\ g^{-1}$ HMA.

The survival of cells in the drug-treated tumours was determined by use of an *in vivo–in vitro* excision assay. At 20–24 h after treatment, mice were killed by cervical dislocation. Tumours were excised, weighed and then minced with scissors in PBS. They were treated with trypsin (100 $\mu g\ ml^{-1}$) and DNAseI (100 $\mu g\ ml^{-1}$) at 37°C for 30 min to obtain single-cell suspensions. The resulting cell suspensions were then passed through a nylon filter to remove undigested clumps of cells, centrifuged and resuspended in PBS. The number of viable cells in the suspension was determined by staining the cells with trypan blue and dye-excluding cells were counted with a haemocytometer. Tumour cell suspensions were diluted and plated in six-well plates (Nuncton, Denmark). After 7 days, the plates were stained and colonies were counted. From the number of cells recovered per gram of tumour and the plating efficiency of the cells, surviving fraction per tumour was calculated.

### Results

### *Inhibition of the $Na^+/H^+$ antiport activity*

In initial experiments, we studied the inhibition of the $Na^+/H^+$ antiport by amiloride, EIPA and HMA in EMT-6 cells using BCECF and fluorometry (Figure 1). From the concentration to give 50% inhibition ($IC_{50}$) of the antiport, HMA and EIPA were about 100 times more potent than amiloride in EMT-6 cells. The $IC_{50}$ of HMA in EMT-6 cells was $\sim$40 nM; and $\geqslant 90\%$ inhibition was observed at concentrations above 1 $\mu M$. In limited experiments to study the effect of $Na^+/H^+$ inhibition on cellular uptake of $^{22}Na^+$ we found that 1 $\mu M$ HMA was as effective as 100 $\mu M$ amiloride in providing $>90\%$ inhibition (data not shown). Similar results were observed in KHT cells (data not shown).

### In vitro *cytotoxicity of HMA in monolayer cell culture*

Previous experiments have suggested that amiloride and EIPA are toxic to cells at low $pH_e$ if cells are first exposed to an agent that causes acidification of the cytoplasm (Maidorn *et al.*, 1993). Therefore, to assess the *in vitro* cytotoxicity of amiloride, EIPA and HMA, survival experiments were performed with varying doses, $pH_e$ values and exposure times in the presence (or absence, for HMA only) of nigericin at 0.25 $\mu g\ ml^{-1}$.

Nigericin caused cell killing only at $pH_e$ below 6.4 (Figure 2). HMA, at a concentration as low as 1 $\mu M$, was able to increase the cytotoxicity of nigericin and to extend the range

**Figure 1** Activity of the $Na^+/H^+$ exchanger of EMT-6 cells exposed to varying concentrations of amiloride (□), EIPA (◆) or HMA (▲). Points, mean of two measurements; error bars, range.

**Figure 2** Relative plating efficiency (RPE) of EMT-6 cells treated with HMA (1 μM) alone (●) or nigericin (0.25 μg $ml^{-1}$) alone (□) or both (○) for 4.5 h in relation to $pH_e$ of the exposure medium. Points, means of measurements from two independent experiments; error bars, range of mean values.

**Figure 3** **a**, Relative plating efficiency (RPE) of EMT-6 cells treated for 4.5 h with different doses of amiloride, EIPA or HMA in the presence (or absence, for HMA only) of 0.25 μg $ml^{-1}$ nigericin at $pH_e$ ~6.5. **b**, Relative plating efficiency (RPE) of EMT-6 cells treated for varying times with a given dose of amiloride, EIPA or HMA in the presence (or absence, for HMA only) of 0.25 μg $ml^{-1}$ nigericin at $pH_e$ ~6.5. Points, means of measurements from two independent experiments; error bars, range of mean values.

of permissive $pH_e$ to ~6.8, while HMA alone failed to achieve any cytotoxicity. Nigericin and HMA, at concentrations of 0.25 μg $ml^{-1}$ and 1μM respectively, showed approximately a 10-fold decrease in cell survival for every 0.15 units decrease of the $pH_e$ in the range of 6.2–6.8 (Figure 2).

The dose–response relationship for cell killing at $pH_e$ 6.5 is shown in Figure 3a. Nigericin alone had a small effect (surviving fraction 0.7). Amiloride, EIPA and HMA gave a dose-dependent increase in the cytotoxicity of nigericin, but EIPA and HMA were about 100 times more potent than amiloride, similar to their relative potencies for inhibition of the $Na^+/H^+$ antiport (Figure 1). Without nigericin, HMA alone gave almost no cell killing (Figure 3a). There was no plateau effect at high concentrations for any of the agents, even though smaller concentrations of HMA and EIPA (1 μM) can achieve effective suppression of the $Na^+/H^+$ exchanger activity (Figure 1).

The relationship between exposure time and cytotoxicity at $pH_e$ ~6.5 is shown in Figure 3b. Nigericin (0.25 μg $ml^{-1}$) alone gave a surviving fraction of ~0.1 after 6 h exposure. When amiloride, EIPA or HMA was used in combination with nigericin, they all increased the cytotoxicity of nigericin for any given exposure time. Time-dependent survival curves were similar for nigericin used with 100 μM amiloride, 1μM EIPA or 1 μM HMA (Figure 3b). Nigericin (0.25 μg $ml^{-1}$) plus 10 μM HMA led to higher levels of cytotoxicity than nigericin (0.25 μg $ml^{-1}$) plus 1 μM HMA, again demonstrating the absence of a plateau effect for cell killing.

*Cell killing in intact and dissociated spheroids*

Further experiments were performed with the potent inhibitor of $Na^+/H^+$ exchange, HMA. To determine whether HMA has good tissue penetration, the cell killing effect of nigericin and HMA was compared in intact and dissociated EMT-6 spheroids. The results of experiments which compare the time course of cell survival in intact spheroids and in cells obtained from their prior dissociation are shown in Figure 4. Cells in intact and dissociated spheroids treated with nigericin alone or with HMA alone showed limited cytotoxicity when exposed to these agents in medium at $pH_e$ 6.45. HMA increased the cytotoxicity of nigericin for both intact spheroids and for cells from dissociated spheroids. Greater cell killing was observed in the dissociated than in the intact spheroids, but, since cell survival after 4.5 h incubation was reduced to $< 10^{-2}$ in intact spheroids treated with nigericin and HMA, both agents can penetrate through layers of tissue to kill the internal cells. To obtain similar level of cytotoxicity to those observed for cells in monolayer culture, higher doses (40 μM) of HMA were required for treatment of spheroids, and for treatment of cells immediately after dissociating spheroids (compare with Figure 3b). Thus, cells in spheroids are more resistant to drug treatment than single cells in culture.

A further experiment was carried out in which cells obtained by dissociating spheroids were plated in Petri dishes for 3 days (until confluent), and then treated with nigericin and HMA. The sensitivity of these cells to drug treatment recovered to a level that was only slightly less than that of the original cultured cells (data not shown).

*Cell killing at different depths in spheroids*

In order to examine drug effects at different depths in spheroids, cells were obtained from drug-treated EMT-6 spheroids (treated at $pH_e$ 6.45 with 50 μM HMA,

**Figure 4** Relative plating efficiency (RPE) of intact (I) or dissociated (D) EMT-6 spheroids treated for varying times with 40 μM HMA or 0.25 μg $ml^{-1}$ nigericin or both drugs at $pH_e$ 6.45. Control samples were treated with diluent. Points, means of measurements from two independent experiments; error bars, range of mean values.

**Figure 5** Relative plating efficiency (RPE) in different fractions of cells from EMT-6 spheroids, stained with Hoechst 33342 and separated by FACS. Fraction 1 represents the inner region (dimmest) and fraction 4 the outer region (brightest). Spheroids were exposed to drugs or diluent for 4.5 h at $pH_e$ 6.45. Columns, means of measurements from two independent experiments; error bars, range of mean values. □, HMA (50 μM); ▨, nigericin (0.25 μg $ml^{-1}$); ■, nigericin + HMA.

0.25 μg $ml^{-1}$ nigericin, alone or in combination) that were exposed to Hoechst 33342. These cells were sorted by FACS into four equal fractions with the same number of cells in each fraction. Fractions 1 (dimmest) to 4 (brightest) represented the cells from inner to outer regions of the spheroids. The ratio of mean Hoechst 33342 fluorescence in the brightest and dimmest fractions was about 18 in the two independent experiments that were used to assess cell survival. Cell recovery from the spheroids after drug treatment was ~88%. The PE of the inner fraction was somewhat lower than the other three fractions, indicating that there were less viable cells in this region (data not shown). In order to correct for this effect, RPEs of the four fractions of drug-treated samples relative to the four corresponding fractions of control samples were calculated and are shown in Figure 5. HMA alone showed little or no cytotoxicity, and nigericin alone caused minimal cytotoxicity in these four fractions. When nigericin was used in combination with HMA, the cytotoxicity in each of the four fractions was markedly increased. There were at most small differences in the level of cell survival in these four fractions, indicating that nigericin and HMA can penetrate through layers of tissue in the spheroids to give similar cell killing at different depths of penetration.

*Pharmacokinetics of HMA in Balb/c mice*

The concentration of HMA in mouse plasma was determined by HPLC. To construct a standard curve, plasma samples containing different concentrations of HMA with the same amount of benzamile added were analysed (Figure 6a). The ratio of the areas under each HMA and benzamile peak was plotted against HMA concentration. The standard curve was linear over the range of HMA concentrations (0.1–50 μM) that might be expected to be present in mouse plasma, and the correlation coefficient was 0.999 (Figure 6a). The sensitivity of the method allows detection of about 0.1 μM HMA in plasma.

The plasma concentration–time curve for HMA in Balb/c mice averaged from two independent experiments is shown in Figure 6b. Immediately after injection of an i.p. dose of 20 μg $g^{-1}$, the concentration of HMA in plasma increased and reached a peak level of 8 μM at about 15 min. Thereafter, the concentration of HMA decreased at a rate that approximates a monoexponential decay. From the curve, it is estimated that the half-life (the time at which half of the initial concentration is reached) of HMA in Balb/c mouse plasma is about 35 min. At about 120 min after injection, the concentration of HMA in plasma decreased to 1 μM, which corresponds to the lowest effective concentration of HMA (when combined with nigericin) which led to cytotoxicity for EMT-6 cells in monolayer culture (see Figure 3a).

**Figure 6** **a**, Standard curve which characterises the quantitation of HMA in Balb/c mouse plasma, as detected by HPLC. The ordinate indicates the ratio of the area under the HMA peak to that under a standard (100 ng of benzamile) peak. **b**, The plasma concentration–time curve of HMA in Balb/c mouse plasma following an i.p. injection of 20 μg $g^{-1}$ HMA. Points, means of measurements from two independent experiments; error bars, range.

*In vivo effects of nigericin and HMA on the EMT-6 tumour*

In preliminary experiments we determined that mice tolerated doses of 2.5 μg $g^{-1}$ nigericin alone, 30 μg $g^{-1}$ HMA alone or a combination of 1.25 μg $g^{-1}$ nigericin and 10 μg $g^{-1}$ HMA. At higher doses death of mice after 24 h was observed (Data not shown).

Multiple experiments were performed to determine the surviving fraction per tumour (SF/tumour) following treatment of mice with either one, two or all three of the drugs hydralazine, nigericin and HMA (Figure 7). When mice bearing EMT-6 tumours were treated with HMA alone, hydralazine alone or two of the three drugs in combination, cytotoxicity was minimal. Only the combination of the three drugs ($10\,\mu g\,g^{-1}$ HMA + $1.25\,\mu g\,g^{-1}$ nigericin plus $10\,\mu g\,g^{-1}$ hydralazine) led to a SF/tumour of less than $10^{-2}$. In comparison with control, the recovery of dye-excluding cells per gram of tumour was about ten times lower following treatment with three drugs in combination. The result of an experiment in which mice were given three injections of nigericin and HMA at 2 h intervals is also shown in Figure 7; multiple doses of nigericin and HMA failed to show significant *in vivo* cytotoxicity. We did not investigate the use of multiple doses in combination with hydralazine.

In further experiments, tumour irradiation was used to select a population of hypoxic and presumably acidic cells. The results of experiments which determined the SF tumour of the above agents used with and without radiation are shown in Figure 8. Radiation (15 Gy) led to a surviving

**Figure 7** Surviving fraction per tumour (SF tumour) for the EMT-6 tumour following the indicated treatments. Columns, means of independent estimates from 3–7 mice; error bars, standard deviations. □, HMA ($10\,\mu g\,g^{-1}$); ▨, hydralazine ($10\,\mu g\,g^{-1}$) + HMA ($10\,\mu g\,g^{-1}$); ▧, hydralazine ($10\,\mu g\,g^{-1}$); ▩, hydralazine ($10\,\mu g\,g^{-1}$) + nigericin ($1.25\,\mu g\,g^{-1}$); ▤, nigericin ($1.25\,\mu g\,g^{-1}$) + HMA ($10\,\mu g\,g^{-1}$); □, three injections (2 h interval) of HMA ($4\,\mu g\,g^{-1}$) + nigericin ($0.45\,\mu g\,g^{-1}$) for each injection; ■, hydralazine ($10\,\mu g\,g^{-1}$) + nigericin ($1.25\,\mu g\,g^{-1}$) + HMA ($10\,\mu g\,g^{-1}$).

**Figure 8** Surviving fraction per tumour (SF tumour) of the EMT-6 tumour following 15 Gy radiation, either alone or with drugs given 30 min after radiation treatment. Columns, means of independent estimates from 3–7 mice; error bars, standard deviation. □, radiation (15 Gy); ▨, radiation (15 Gy) + HMA ($10\,\mu g\,g^{-1}$); ▧, radiation (15 Gy) + hydralazine ($10\,\mu g\,g^{-1}$); ▩, radiation (15 Gy) + hydralazine ($10\,\mu g\,g^{-1}$) + nigericin ($1.25\,\mu g\,g^{-1}$); ▤, radiation (15 Gy) + hydralazine ($10\,\mu g\,g^{-1}$) + HMA ($10\,\mu g\,g^{-1}$); □, radiation (15 Gy) + nigericin ($1.25\,\mu g\,g^{-1}$) + HMA ($10\,\mu g\,g^{-1}$); ■, radiation (15 Gy) + hydralazine ($10\,\mu g\,g^{-1}$) + nigericin ($1.25\,\mu g\,g^{-1}$) + HMA ($10\,\mu g\,g^{-1}$).

fraction of $\sim 10^{-2}$. When radiation was followed by $10\,\mu g\,g^{-1}$ HMA there was no significant increase in cell killing. When radiation was followed by hydralazine, or by the combinations of two of the three drugs (e.g. nigericin + HMA), there was a reduction in cell survival to about $2\times10^{-3}$. The toxicity of radiation followed by all three of the drugs in combination (hyralazine, HMA and nigericin) decreased the level of cell survival to about $10^{-4}$. However, the effect of these drugs used in combination with radiation was no greater than the sum of the effect of the three drugs in combination and that of radiation alone (additive model).

### Discussion

Under the microenvironmental conditions found within the acidic regions of solid tumours, the $Na^+$ $H^+$ antiport was found to be an important and perhaps dominant mechanism for regulation of $pH_i$ (Boyer & Tannock, 1992). Function of the antiport also appears to be required for the growth of at least one type of solid tumour (Rotin *et al.*, 1989). Previous experiments have shown that amiloride, in combination with cell-acidifying agents (e.g. nigericin, CCCP), causes cytotoxicity at low $pH_e$ in tissue culture (Rotin *et al.*, 1987; Newell & Tannock, 1989). Furthermore, EIPA and other potent amiloride analogues can cause a higher level of cytotoxicity than amiloride (Maidorn *et al.*, 1993). The present study demonstrates that HMA and EIPA increased the cytotoxicity of nigericin to a similar extent: both are $\sim 100$ times more potent than amiloride in causing $pH_e$-dependent cytotoxicity (Figures 2 and 3). The relative potency of amiloride analogues in causing cytotoxicity correlates with their relative potency in inhibition of the $Na^+$ $H^+$ antiport (Figure 1), suggesting that inhibition of the $Na^+$ $H^+$ antiport, rather than other non-specific effects, is largely responsible for the cytotoxicity of these agents at low $pH_e$. More convincing evidence came from the study of Maidorn *et al.* (1993) in which EIPA did not increase the cytotoxicity of nigericin in a mutant cell line, PS-120, which lacks the $Na^+$ $H^+$ antiport.

The cell-acidifying agent nigericin causes cytotoxicity only under condition of $pH_e$ below 6.5. HMA extended the permissive range of $pH_e$ to $<6.8$ (Figure 2). *In vivo*, estimates of $pH_e$ below 6.8 have been recorded in solid tumours, but not in most normal tissues. This result provides the basis for the potential selectivity of HMA in killing cells within the acidic microenvironment of solid tumours.

Although the concentrations of HMA and EIPA required to give maximal inhibition of $Na^+$ $H^+$ antiport activity were about 1 $\mu$M, there was a continuous fall in cell survival within the dose range of HMA and EIPA tested (Figure 3a). Failure to observe a maximum level of cell killing may occur because the survival of cells was assessed after a 4.5 h exposure to nigericin and HMA, whereas inhibition of the $Na^+$ $H^+$ antiport was determined over a period of several minutes (Figures 1–3). Also, the experiments which quantitate inhibition of $Na^+$ $H^+$ exchange activity (Figure 1) might be insensitive to small changes within the range of 90–100% inhibition, and we cannot exclude some additional toxic effects due to other non-specific mechanisms.

The potential of nigericin and HMA to cause anti-tumour effects *in vivo* depends on their ability to penetrate tissue, and this effect was studied in spheroids. Although there was greater cell killing in dissociated spheroids than in intact ones, a high level of cell killing in intact spheroids was still observed, suggesting that both agents can penetrate through layers of tissue to kill the inner cells. The possible reasons for failing to observe the same level of cell killing in intact and dissociated spheroids include: (a) cells from dissociated spheroids and cells in intact spheroids may have different sensitivity to the drugs because of factors related to cell contact or to the microenvironment; (b) dissociation of spheroids using trypsin and gentle mechanical disaggregation may make the cells more sensitive to the drug treatment; and or (c) there may be minor problems with drug penetration, such that the average concentration of drugs in the central region of intact spheroids is lower than in the outer regions.

Higher concentrations of HMA (40–50 $\mu$M) were required to achieve similar cytotoxicity in intact spheroids or cells immediately dissociated from spheroids as compared with cells maintained in monolayer culture (1–10 $\mu$M) (compare Figures 3 and 4). Thus, cells in EMT-6 spheroids and cells immediately dissociated from them are more resistant to drug treatment. Additional experiments (data not shown) revealed that cells first dissociated from spheroids and then plated for 3 days (until confluent) became almost as sensitive to the drug treatment as the original cultured cells. Spheroids have been reported to be more resistant to treatments with ionising radiation, heat, ultrasound and doxorubicin (Dertinger & Huesler, 1981; Durand, 1981; Sacks *et al.*, 1981; Wigle & Sutherland, 1985). Resistance to ionising radiation in Chinese hamster V79 lung cells was found to persist for about one cell cycle (10 h) after dissociation of the spheroids (Durand & Sutherland, 1972). Sutherland (1988) suggested that a history of growth in close cell–cell contact is the most important factor for such phenomena, and that direct cell–cell communication at the time of treatment may not be critical for the 'contact effect'. For treatment with agents that act on the cell surface such as nigericin and HMA, cell contact might inhibit the effects of drugs to cause acidification (nigericin) or limit access to $Na^+/H^+$ antiport proteins (HMA). Relative resistance might also occur if the activity of these drugs was dependent on cell proliferation, since many cells in spheroids may be out of cycle.

Since the above method measured only the average cell killing in spheroids, fluorescence-activated cell sorting (FACS) was used to determine cytotoxicity as a function of depth throughout the spheroids. A high concentration of HMA (50 $\mu$M) was used in such experiments because of resistance of spheroids to the drug treatment. Only minor differences in cell survival were observed in the four sorted fractions (Figure 5), suggesting that HMA can penetrate through spheroids and can give similar cell killing at different depths. However, because of spherical geometry, the sorting of cells from spheroids into four equal fractions does not rule out minor problems of penetration to the deepest viable cells. Spheroids used in experiments had a mean diameter of $\sim$600 $\mu$m, and would have a necrotic centre of diameter $\sim$200 $\mu$m. Separation of the viable regions into four equal fractions then leads to shells of thickness of $\sim$190 $\mu$m (innermost), $\sim$90 $\mu$m, $\sim$65 $\mu$m and $\sim$55 $\mu$m (outermost). Variations in sensitivity to the drugs in the innermost shell cannot be excluded by this method. When spheroids are grown at physiological pH, there is evidence for a lower $pH_e$ in the central region (Carlsson & Acker 1988). We were not able to measure $pH_e$ in our spheroids, but it is possible that central regions had lower $pH_e$ than peripheral regions even when the $pH_e$ of the medium was $\sim$6.5. Thus, failure to observe differences in cell killing among the sorted fractions might have been due to opposing effects of (i) a modest barrier to penetration and (ii) increased sensitivity of internal cells.

Pharmacokinetic studies are required to derive strategies to deliver and maintain a desirable concentration of HMA *in vivo*. Accordingly, a sensitive HPLC method was developed to quantitate plasma levels of HMA in the Balb/c mouse. The plasma concentration–time curve of HMA (Figure 6b) showed that the peak concentration was about 8 $\mu$M after i.p. administration of a single dose of 20 $\mu g\ g^{-1}$. The elimination half-life was estimated as 35 min. The plasma concentration of HMA was reduced below 1 $\mu$M after approximately 120 min. These results, in conjunction with previous results from this laboratory (Newell *et al.*, 1992) which showed reduction of $pH_i$ *in vivo* within 2 h after i.p. injection of nigericin, suggest that *in vivo* killing of cells is feasible if cells *in vivo* have similar sensitivity to those in monolayer culture. Greater cell killing at tolerated doses might be achieved by delivering drugs by constant infusion to maintain plasma levels at a constant value for several hours.

The present study showed that HMA and nigericin could achieve effective cell killing below $pH_e$ 6.8 *in vitro*. Newell *et al.* (1992) reported that the average $pH_e$ of EMT-6 tumours grown in the legs of mice is about 6.75 (about 0.3 pH units below muscle $pH_e$); $pH_e$ is expected to depend on location relative to blood vessels, with lower $pH_e$ at increasing distance from blood vessels. However, cytotoxicity was not observed when single or multiple doses of HMA and nigericin were administered *in vivo* (Figure 7). The failure to observe cytotoxicity probably occurs because at doses of HMA and nigericin which can be achieved *in vivo* the $pH_e$ in most regions of tumours is too high to obtain selective cell killing. It is probable that spheroids provide a better model for tumours than single cells in culture, and that relative drug resistance may be expected from cells in contact in the *in vivo* environment.

It has been shown that hydralazine can cause a reduction in tumour blood flow and oxygen delivery (Lin & Song, 1990; Chaplin *et al.*, 1991; Peters & Chaplin, 1992). The effect is thought to occur because blood vessels in normal tissues dilate, whereas those in tumours lack smooth muscle and consequently will not respond to agents which act on vascular smooth muscle. We and others have found that hydralazine may lead to a modest reduction in mean $pH_e$ and/or $pH_i$ of tumours (Okunieff *et al.*, 1989). Hydralazine was found to potentiate the cytotoxicity of nigericin and HMA in EMT-6 tumours (Figure 7), and values of SF/tumour were reduced to below $10^{-2}$. The possible mechanisms for such potentiation include the following:

1. reduction in tumour $pH_e$ leading to increased cytotoxicity of these acid-dependent agents;
2. alteration of drug pharmacokinetics;
3. direct interaction of hydralazine with HMA and nigericin to potentiate cytotoxicity;
4. combined effects of hypoxia and reduced $pH_e$ to enhance cell killing by drugs.

The use of a high concentration of hydralazine (50 $\mu g\ ml^{-1}$) does not increase the cytotoxicity of nigericin and EIPA *in vitro* (K. Hasuda & I. Tannock, personal communication), indicating that a direct interaction is unlikely to be the mechanism responsible for the potentiation of cytotoxicity observed *in vivo*. Rotin *et al.* (1986) reported that the combination of hypoxia and acidity could cause higher cytotoxicity to cells than acidity alone, probably because both factors lead to ATP depletion. Thus, the combination of hypoxia and acidity caused by hydralazine could be a mechanism responsible for the observed potentiation of nigericin and HMA (Figure 7). Consistent with the above interpretation, experiments in our laboratory have shown that glucose injection caused a greater reduction in tumour $pH_e$ than administration of hydralazine, but was less effective in potentiating the cytotoxicity of nigericin and EIPA than hydralazine (K. Hasuda & I. Tannock, personal communication). Hydralazine may cause both hypoxia and reduced $pH_e$, whereas glucose acts mainly to reduce $pH_e$. Finally, a vasoactive agent such as hydalazine will influence the delivery and removal of drugs to and from the tumour. It is probable that multiple mechanisms are responsible for the potentiation by hydralazine of the cytotoxicity of nigericin plus HMA.

Since hypoxic and acidic subpopulations of tumour cells are likely to be more sensitive to drug treatment, experiments were also performed with radiation (before drug treatment) to deplete aerobic and putatively less acidic tumour cells. The toxic effects of hydralazine, nigericin and HMA appeared to be additive with those of radiation and do not suggest selective toxicity towards cells in the hypoxic/acidic microenvironments of solid tumours. This effect might occur because significant toxicity of nigericin and HMA requires the presence of hydralazine, which even in the absence of radiation enlarges the hypoxic and acidic subpopulations; in effect, both hydralazine and radiation may be selecting a hypoxic and acidic subpopulation. It is also possible that the subpopulations of hypoxic cells and acidic cells in tumours are discrete, since hypoxia may occur adjacent to regions of necrosis, whereas necrotic regions of tumours have been reported to be slightly alkaline (Kallinowski & Vaupel, 1988).

Acute hypoxia may also occur in non-acid regions owing to rapid fluctuations in blood flow. These effects would imply the potential for therapeutic benefits from combining agents that are selectively toxic under hypoxic and under acidic conditions.

The results of the present study indicate that HMA, in combination with nigericin and hydralazine, can lead to death of cells in solid tumours. This effect is probably due in part to inhibition of regulation of $pH_i$ under the environmental conditions that occur in solid tumours. Additional strategies which maximise the $pH_e$ differential between tumours and normal tissues, and which inhibit bicarbonate-based mechanisms of $pH_i$ regulation, may allow the development of acid-mediated selective tumour therapy.

**Abbreviations**

BCECF-AM, 2', 7'-bis-(2-carboxyethyl)-5-(and 6)-carboxyfluorescein acetoxymethylester; CCCP, carbonylcyamide-3-chlorophenylhydrazone; EIPA, 5-(*N*-ethyl-*N*-isopropyl) amiloride; FACS, fluorescence-activated cell sorting; HMA, 5-(*N,N*-hexamethylene) amiloride; HPLC, high-performance liquid chromatography; $IC_{50}$, half-maximal inhibition concentration; i.p., intraperitoneal; LIGF, log integrated green fluorescence; NMG, *N*-methyl-D-glucamine; PBS, phosphate-buffered saline; PE, plating efficiency, $pH_e$, extracellular pH; $pH_i$ intracellular pH; RPE, relative plating efficiency; SF tumour: survival fraction per tumour; α-MEM, α-minimal essential medium.

This study was supported by a research grant from the Medical Research Council of Canada.

**References**

ALLIEGRO, M.A., DYER, K.D., CRAGOE, Jr. E.J., GLASER, B.M. & ALLIEGRO M.C. (1992). High-performance liquid chromatographic method for quantitating plasma levels of amiloride and its analogues. *J. Chromatogr.*, **582**, 217–223.

BOYER, M.J. & TANNOCK, I.F. (1992). Regulation of intracellular pH in tumor cell lines: influence of microenvironmental conditions. *Cancer Res.*, **52**, 4441–4447.

BUSA, W.B. & NUCCITELLI, R. (1984). Metabolic regulation via intracellular pH. *Am. J. Physiol.*, **246**, R409–R438.

CARLSSON, J. & ACKER, H. (1988). Relations between pH, oxygen partial pressure and growth in cultured spheroids. *Int. J. Cancer*, **42**, 715–720.

CASSEL, D., SCHARF, O., ROTMAN, M., CRAGOE, Jr. E.J. & KATZ, M. (1988). Characterization of $Na^+$-linked and $Na^+$-independent $Cl^-$ $HCO^-_3$ exchange systems in Chinese hamster lung fibroblasts. *J. Biol. Chem.*, **263**, 6122–6127.

CHAPLIN, D.J., PETERS, C.E., HORSMAN, M.R. & TROTTER, M.J. (1991). Drug induced pertubations in tumour blood flow: therapeutic potential and possible limitations. *Radiother. Oncol.*, **20** (Suppl.) 93–101.

CRAGOE, Jr. E.J., WOLTERDORF, Jr. O.W., BICKING, J.B., KWONG, S.F. & JONES, J.H. (1967). Pyrazine diuretics. II. N-amidino-3-amino-5-substituted 6-halopyrazinecarboxamides. *J. Med. Chem.*, **10**, 66–75.

DERTINGER, H. & HUELSER, D. (1981). Increased radioresistance of cells in cultured multicell spheroids. I. Dependence on cellular interaction. *Radiat. Environ. Biophys.*, **19**, 101–107.

DURAND, R.E. (1981). Flow cytometry studies of intracellular adriamycin in multicell spheroids *in vitro*. *Cancer Res.*, **41**, 3495–3498.

DURAND, R.E. (1986). Chemosensitivity testing in V79 spheroids: drug delivery and cellular microenvironment. *J Natl Cancer Inst.*, **77**, 247–252.

DURAND, R.E. (1990). Cisplatin and CCNU synergism in spheroid cell subpopulations. *Br. J. Cancer*, **62**, 947–953.

DURAND, R.E. & SUTHERLAND, R.M. (1972). Effects of intracellular contact on repair of radiation damage. *Exptl. Cell Res.*, **71**, 75–80.

GRINSTEIN, S., ROTIN, D. & MASON, M.J. (1989). $Na^+$ $H^+$ exchange and growth factor-induced cytosolic pH changes. Role in cellular proliferation. *Biochim. Biophys. Acta*, **988**, 73–97.

HORSMAN, M.R., CHAPLIN, D.J. & OVERGAARD, J. (1991). The use of blood flow modifiers to improve the treatment response of solid tumours. *Radiother. Oncol.*, **20**, 47–52.

KALLINOWSKI, F. & VAUPEL, P. (1988). pH distributions in spontaneous and isotransplanted rat tumours. *Br. J. Cancer*, **58**, 314–321.

KALMUS, J., OKUNIEFF, P. & VAUPEL, P. (1990). Dose-dependent effects of hydralazine on microcirculatory function and hyperthermic response of murine FSaII tumors. *Cancer Res.*, **50**, 15–19.

KLEYMAN, T.R. & CRAGOE, Jr. E.L. (1988). Amiloride and its analogs as tools in the study of ion transport. *J. Membrane Biol.*, **105**, 1–21.

L'ALLEMAIN, G., FRANCHI, A., CRAGOE, Jr. E.J. & POUYSSEGUR, J. (1984). Blockade of the $Na^+$ $H^+$ antiport abolishes growth factor-induced DNA synthesis in fibroblasts. *J. Biol. Chem.*, **259**, 4313–4319.

LIN, J.-C. & SONG, C.W. (1990). Effects of hydralazine on the blood flow in RIF-1 tumors and normal tissues of mice. *Radiat. Res.*, **124**, 171–177.

MAIDORN, R.P., CRAGOE, Jr. E.J. & TANNOCK, I.F. (1993). Therapeutic potential of analogues of amiloride: inhibition of the regulation of intracellular pH as a possible mechanism of tumour selective therapy. *Br. J. Cancer*, **67**, 297–303.

MENG, Q.C., CHEN, Y.F. & OPARIL, S. (1990). High-performance liquid chromatographic determination of amiloride and its analogues in rat plasma. *J. Chromatogr.*, **529**, 201–209.

MUSGROVE, E., RUGG, C. & HEDLEY, D. (1986). Flow cytometric measurement of cytoplasmic pH: a critical evaluation of available fluorochromes. *Cytometry*, **7**, 347–355.

NEWELL, K.J. & TANNOCK, I.F. (1989). Reduction of intracellular pH as a possible mechanism for killing cells in acidic regions of solid tumors: effects of carbonylcyanide-3-chlorophenylhydrazone. *Cancer Res.*, **49**, 4477–4482.

NEWELL, K., WOOD, P., STRATFORD, I. & TANNOCK, I.F. (1992). Effects of agents which inhibit the regulation of intracellular pH on murine solid tumours. *Br. J. Cancer*, **66**, 331–317.

OKUNIEFF, P., WALSH, C.S., VAUPEL, P., KALLINOWSKI, F., HITZIG, B.M., NEURINGER, L.J. & SUIT, H.D. (1989). Effects of hydralazine on *in vivo* tumour energy metabolism, hematopoietic radiation sensitivity, and cardiovascular parameters. *Int. J. Radiat. Oncol. Biol. Phys.*, **16**, 1145–1148.

PETERS, C.E. & CHAPLIN, D.J. (1992). Blood flow modification in the SCCVII tumor: effects of 5-hydroxytryptamine, hydralazine, and propranolol. *Int. J. Radiat. Oncol. Biol. Phys.*, **22**, 463–465.

REINERTSEN, K.V., TØNNESSEN, T.I., JACOBSEN, J., SANDVIG, K. & OLSNES, S. (1988). Role of chloride-bicarbonate antiport in the control of cytosolic pH: cell-line differences in activity and regulation of antiport. *J. Biol. Chem.*, **263**, 11117–11125.

RINK, T.J., TSIEN, R.Y. & POZZAN, T. (1982). Cytoplasmic pH and free $Mg^{2+}$ in lymphocytes. *J. Cell Biol.*, **95**, 189–196.

ROTIN, D. & GRINSTEIN, S. (1989). Impaired cell volume regulation in $Na^+$ $H^+$ exchange deficient mutants. *Am. J. Physiol.*, **257**, C1158–1165.

ROTIN, D., ROBINSON, B. & TANNOCK, I.F. (1986). Influence of hypoxia and an acidic environment on the metabolism and viability of cultured cells: potential implications for cell death in tumors. *Cancer Res.*, **46**, 2821–2826.

ROTIN, D., WAN, P., GRINSTEIN, S. & TANNOCK, I.F. (1987). Cytotoxicity of compounds that interfere with the regulation of intracellular pH: a potential new class of anticancer drugs. *Cancer Res.*, **47**, 1497–1504.

ROTIN, D., STEELE-NORWOOD, D., GRINSTEIN, S. & TANNOCK, I. (1989). Requirements of the $Na^+$ $H^+$ exchanger for tumor growth. *Cancer Res.*, **49**, 205–211.

SACKS, P.G., MILLER, M.W. & SUTHERLAND, R.M. (1981). Influences of growth conditions and cell-cell contact on responses of tumour cells to ultrasound. *Radiat. Res.*, **87**, 175–186.

SIMCHOWITZ, L. & CRAGOE, Jr. E.J. (1986). Inhibition of chemotactic factor-activated $Na^+$/$H^+$ exchange in human neutrophils by analogues of amiloride: structure–activity relationships in the amiloride series. *Mol. Pharmacol.*, **30**, 112–120.

SUTHERLAND, R.M. (1988). Cell and environment interactions in tumor microregions: the multicell spheroid model. *Science*, **240**, 177–184.

VAUPEL, P., KALLINOWSKI, F. & OKUNIEFF, P. (1989). Blood flow, oxygen and nutrient supply, and metabolic microenvironment of human tumors: a review. *Cancer Res.*, **49**, 6449–6465.

WIGLE, J.C. & SUTHERLAND, R.M. (1985). Increased thermoresistance developed during growth of small multicellular spheroids. *J. Cell. Physiol.*, **122**, 281–289.

WIKE-HOOLEY, J.L., HAVEMAN, J. & REINHOLD, H.S. (1984). The relevance of tumour pH to the treatment of malignant disease. *Radiother. Oncol.*, **2**, 343–366.